# Supplementary material for: Analysis-ready datasets for insecticide resistance phenotype and genotype frequency in African malaria vectors
Source: Sci Data. 2019 Jul 15;6:121. doi: 10.1038/s41597-019-0134-2 (PMC6629700; doi:10.1038/s41597-019-0134-2)
Supplement: Supplementary file 2 — Supplementary Information. [file 41597_2019_134_MOESM2_ESM.pdf]

Supplementary Information

Inclusion criteria 2 - 3

Recommended doses and exposure periods 4 - 8

Species identification methods 9 - 29

Inclusion criteria for data on the prevalence of the insecticide resistance phenotype

1. Was a WHO susceptibtiliy test used?
    a. If yes, was 100% mortality in the susceptible strain acheived after exposure to the treated paper?
        i. If yes, see question 2.
        ii. If no, exclude.
    b. If no, was a CDC bottle bioassay used?
        i. If yes, see question 2.
        ii. If no, exclude.
2. Were F0 or F1 generation mosquitoes derived from a field collection used?
    a. If yes, see question 3.
    b. If no, exclude.
3. Are the results disaggregated to species?
    a. If no, include in the dataset for the relevant complex/subgroup only.
    b. If yes, can the species results be combined to provide an unbiased result for the original complex/subgroup sample?
        i. If yes, include each species result in the species dataset and include the combined result in the complex/subgroup dataset.
        ii. If no, include each species result in the species dataset only.

Inclusion criteria for data on *Vgsc* allele frequencies

1. Were *Vgsc* alleles tested for?
    a. If yes, see question 2.
    b. If no, exclude.
2. Were F0 or F1 generation mosquitoes derived from a field collection used?
    a. If yes, see question 3.
    b. If no, exclude.
3. Are the results disaggregated to species and/or to dead/alive mosquitoes?
    a. If no, include in the dataset for the relevant complex/subgroup only.
    b. If disaggregated to species only, can the species results be combined to provide an unbiased result for the original complex/subgroup sample?
        i. If yes, include each species result in the species dataset and include the combined result in the complex/subgroup dataset.
        ii. If no, include each species result in the species dataset only.
    c. If disaggregated to dead/alive mosquitoes only, can the dead/alive results be combined to provide an unbiased result for the original complex/subgroup sample?
        i. If yes, include in the dataset for the relevant complex/subgroup.
        ii. If no, exclude.
    d. If disaggregated to both species and to dead/alive, can the dead/alive results for a species be combined to provide an unbiased result for that species?
        i. If no, exclude.

ii. If yes, can the species results then be combined to provide an unbiased result for the original complex/subgroup sample?
    I. If yes, include each species result in the species dataset and include the combined result in the complex/subgroup dataset.
    II. If no, include each species result in the species dataset only.

	DDT		Dieldrin	
**Protocol**	**Concentration**	**Duration (min.)**	**Concentration**	**Duration (min.)**
**WHO 1963**	multiple	60	multiple	60
**WHO 1970**	multiple	60	multiple	60
**WHO 1975**	multiple	60	multiple	60
**WHO 1976**	multiple	60	multiple	60
**WHO 1980**	4%	60	4%	60
**WHO 1981**	4%	60	4%	60
**WHO 1982**	4%	60	4%	60
**WHO 1986**	4%	60	4%	60
**WHO 1988**	4%	60	4%	60
**WHO 1992**	4%	60	4%	60
**WHO 1998**	4%	60	4%	60
**WHO 2013**	4%	60	0.4%, 4%	60
**WHO 2016**	4%	60	4%	60
**CDC**	100 µg/bottle	45		

	Fenthion		Propoxur	
**Protocol**	**Concentration**	**Duration (min.)**	**Concentration**	**Duration (min.)**
**WHO 1963**				60
**WHO 1970**	0.25%, 2.5%	60	0.01%, 0.1%	60
**WHO 1975**	0.25%, 2.5%	60	0.01%, 0.1%	60
**WHO 1976**	0.25%, 2.5%	60	0.01%, 0.1%	60
**WHO 1980**			0.1%	60
**WHO 1981**			0.1%	60
**WHO 1982**			0.1%	60
**WHO 1986**	2.5%	60	0.1%	60
**WHO 1988**			0.1%	60
**WHO 1992**			0.1%	60
**WHO 1998**			0.1%	60
**WHO 2013**			0.1%	60
**WHO 2016**			0.1%	60
**CDC**				

	Malathion		Fenitrothion	
**Protocol**	**Concentration**	**Duration (min.)**	**Concentration**	**Duration (min.)**
**WHO 1963**	0.5%, 5%	60	0.1%, 1%	60
**WHO 1970**	0.5%, 5%	60	0.1%, 1%	60
**WHO 1975**	0.5%, 5%	60	0.1%, 1%	60
**WHO 1976**	0.5%, 5%	60	0.1%, 1%	60
**WHO 1980**	5%	60	1%	120
**WHO 1981**	5%	60	1%	120
**WHO 1982**	5%	60	1%	120
**WHO 1986**	5%	60	1%	120
**WHO 1988**	5%	60	1%	120
**WHO 1992**	5%	60	1%	120
**WHO 1998**	5%	60	1%	120
**WHO 2013**	5%	60	1%	120
**WHO 2016**	5%	60	1%	120
**CDC**	50 µg/bottle	30	50 µg/bottle	30

	Chlorphoxim		Permethrin	
**Protocol**	**Concentration**	**Duration (min.)**	**Concentration**	**Duration (min.)**
**WHO 1963**				
**WHO 1970**				
**WHO 1975**				
**WHO 1976**				
**WHO 1980**	4%	60	0.25%	60
**WHO 1981**	4%	60	0.25%	60
**WHO 1982**			0.25%	60
**WHO 1986**			0.25%	60
**WHO 1988**			0.25%	60
**WHO 1992**			0.25%	60
**WHO 1998**			0.75%	60
**WHO 2013**			0.75%	60
**WHO 2016**			0.75%	60
**CDC**			21.5 µg/bottle	30

	Deltamethrin		Lambda-cyhalothrin	
**Protocol**	**Concentration**	**Duration (min.)**	**Concentration**	**Duration (min.)**
**WHO 1963**				
**WHO 1970**				
**WHO 1975**				
**WHO 1976**				
**WHO 1980**	0.025%	60		
**WHO 1981**	0.025%	60		
**WHO 1982**	0.025%	60		
**WHO 1986**	0.025%	60		
**WHO 1988**	0.025%	60		
**WHO 1992**	0.025%	60	0.1%	60
**WHO 1998**	0.5%	60	0.05%	60
**WHO 2013**	0.5%	60	0.05%	60
**WHO 2016**	0.5%	60	0.05%	60
**CDC**	12.5 µg/bottle	30	12.5 µg/bottle	30

	Pirimiphos-methyl		Carbosulfan	
**Protocol**	**Concentration**	**Duration (min.)**	**Concentration**	**Duration (min.)**
**WHO 1963**				
**WHO 1970**				
**WHO 1975**				
**WHO 1976**				
**WHO 1980**				
**WHO 1981**				
**WHO 1982**				
**WHO 1986**				
**WHO 1988**				
**WHO 1992**				
**WHO 1998**				
**WHO 2013**	0.25%	60	0.40%	60
**WHO 2016**	0.25%	60	0.40%	60
**CDC**	20 µg/bottle	30		

	Bendiocarb		Etofenprox	
**Protocol**	**Concentration**	**Duration (min.)**	**Concentration**	**Duration (min.)**
**WHO 1963**				
**WHO 1970**				
**WHO 1975**				
**WHO 1976**				
**WHO 1980**				
**WHO 1981**	0.1%	60		
**WHO 1982**				
**WHO 1986**				
**WHO 1988**				
**WHO 1992**			0.25%	60
**WHO 1998**	0.1%	60	0.5%	60
**WHO 2013**	0.1%	60	0.5%	60
**WHO 2016**	0.1%	60	0.5%	60
**CDC**	12.5 µg/bottle	30		

	Cyfluthrin		Chlorfenapyr	
**Protocol**	**Concentration**	**Duration (min.)**	**Concentration**	**Duration (min.)**
**WHO 1963**				
**WHO 1970**				
**WHO 1975**				
**WHO 1976**				
**WHO 1980**				
**WHO 1981**				
**WHO 1982**				
**WHO 1986**				
**WHO 1988**				
**WHO 1992**				
**WHO 1998**				
**WHO 2013**	0.15%	60	5%	60
**WHO 2016**	0.15%	60		
**CDC**	12.5 µg/bottle	30		

	Fipronil		Alpha-cypermethrin	
**Protocol**	**Concentration**	**Duration (min.)**	**Concentration**	**Duration (min.)**
**WHO 1963**				
**WHO 1970**				
**WHO 1975**				
**WHO 1976**				
**WHO 1980**				
**WHO 1981**				
**WHO 1982**				
**WHO 1986**				
**WHO 1988**				
**WHO 1992**				
**WHO 1998**				
**WHO 2013**	2%	60		
**WHO 2016**	2%	60	0.05%	60
**CDC**			12.5 µg/bottle	30

	Piperonyl butoxide	
**Protocol**	**Concentration**	**Duration (min.)**
**WHO 1963**		
**WHO 1970**		
**WHO 1975**		
**WHO 1976**		
**WHO 1980**		
**WHO 1981**		
**WHO 1982**		
**WHO 1986**		
**WHO 1988**		
**WHO 1992**		
**WHO 1998**		
**WHO 2013**		
**WHO 2016**	4%	60
**CDC**		

Species	Complex	Subgroup	Group	Series
An. amharicus	gambiae			pyretophorus
An. arabiensis	gambiae			pyretophorus
An. bwambae	gambiae			pyretophorus
An. coluzzii	gambiae			pyretophorus
An. gambiae s.s.	gambiae			pyretophorus
An. coluzzii/gambiae	gambiae			pyretophorus
An. melas	gambiae			pyretophorus
An. merus	gambiae			pyretophorus
An. quadriannulatus	gambiae			pyretophorus
An. nili	nili		ardensis	neomyzomyia
An. carnevalei	nili		ardensis	neomyzomyia
An. ovengensis	nili		ardensis	neomyzomyia
An. somalicus	nili		ardensis	neomyzomyia
An. aruni		funestus	funestus	myzomyia
An. confusus		funestus	funestus	myzomyia
An. funestus		funestus	funestus	myzomyia
An. funestus-like		funestus	funestus	myzomyia
An. longipalpis type C		funestus	funestus	myzomyia
An. parensis		funestus	funestus	myzomyia
An. vaneedeni		funestus	funestus	myzomyia
An. brucei		rivulorum	funestus	myzomyia
An. fuscivenosus		rivulorum	funestus	myzomyia
An. rivulorum		rivulorum	funestus	myzomyia
An. rivulorum-like		rivulorum	funestus	myzomyia
An. leesoni		minimus	funestus	myzomyia
An. moucheti				myzomyia
An. bervoetsi				myzomyia

First Author	Scott	Fanello	Favia	Favia
**Year of publication**	**1993**	**2002**	**1997**	**2001**
**Technique**	PCR: rDNA IGS	PCR-RFLP: rDNA IGS	PCR-RFLP: rDNA IGS	PCR: rDNA IGS
**Species**				
An. amharicus				
An. arabiensis	1	1		
An. bwambae				
An. coluzzii		1	1	1
An. gambiae s.s.		1	1	1
An. coluzzii/gambiae	1			
An. melas	1	1		
An. merus	1	1		
An. quadriannulatus	1	1		
An. nili				
An. carnevalei				
An. ovengensis				
An. somalicus				
An. aruni				
An. confusus				
An. funestus				
An. funestus-like				
An. longipalpis type C				
An. parensis				
An. vaneedeni				
An. brucei				
An. fuscivenosus				
An. rivulorum				
An. rivulorum-like				
An. leesoni				
An. moucheti				
An. bervoetsi				

First Author	Santolamazza	Santolamazza	Wilkins
**Year of publication**	**2008**	**2004**	**2006**
**Technique**	SINE-PCR: SINE200 retrotransposons	PCR-RFLP: rDNA IGS	IMP-PCR
**Species**			
An. amharicus			
An. arabiensis		1	1
An. bwambae			
An. coluzzii	1	1	1
An. gambiae s.s.	1	1	1
An. coluzzii/gambiae			
An. melas			
An. merus			1
An. quadriannulatus			1
An. nili			
An. carnevalei			
An. ovengensis			
An. somalicus			
An. aruni			
An. confusus			
An. funestus			
An. funestus-like			
An. longipalpis type C			
An. parensis			
An. vaneedeni			
An. brucei			
An. fuscivenosus			
An. rivulorum			
An. rivulorum-like			
An. leesoni			
An. moucheti			
An. bervoetsi			

First Author	della Torres	Fanello	Fettene	Fettene
**Year of publication**	**2001**	**2003**	**2003**	**2002**
**Technique**	PCR-RFLP: rDNA IGS	PCR: rDNA IGS	PCR: rDNA IGS	PCR: rDNA IGS
**Species**				
An. amharicus			1	1
An. arabiensis			1	1
An. bwambae				
An. coluzzii	1	1		
An. gambiae s.s.	1	1		
An. coluzzii/gambiae			1	1
An. melas			1	1
An. merus			1	1
An. quadriannulatus			1	1
An. nili				
An. carnevalei				
An. ovengensis				
An. somalicus				
An. aruni				
An. confusus				
An. funestus				
An. funestus-like				
An. longipalpis type C				
An. parensis				
An. vaneedeni				
An. brucei				
An. fuscivenosus				
An. rivulorum				
An. rivulorum-like				
An. leesoni				
An. moucheti				
An. bervoetsi				

First Author	Mahon	Bass	Van Rensburg	
Year of publication	1976	2008	1996	
Technique	Electrophoresis: allozymes	rtPCR	PCR: rDNA IGS	
Species				
An. amharicus				
An. arabiensis	1	1	1	
An. bwambae				
An. coluzzii				
An. gambiae s.s.				
An. coluzzii/gambiae	1	1	1	
An. melas			1	
An. merus	1		1	
An. quadriannulatus	1		1	
An. nili				
An. carnevalei				
An. ovengensis				
An. somalicus				
An. aruni				
An. confusus				
An. funestus				
An. funestus-like				
An. longipalpis type C				
An. parensis				
An. vaneedeni				
An. brucei				
An. fuscivenosus				
An. rivulorum				
An. rivulorum-like				
An. leesoni				
An. moucheti				
An. bervoetsi				

First Author	Miles	Petrarca	Coluzzi
**Year of publication**	**1979**	**1983**	**1967**
**Technique**	Electrophoresis: allozymes		Cytogenetics: salivary glands
**Species**			
An. amharicus			
An. arabiensis			1
An. bwambae	1		
An. coluzzii			
An. gambiae s.s.			
An. coluzzii/gambiae			1
An. melas	1		1
An. merus	1		
An. quadriannulatus	1		
An. nili			
An. carnevalei			
An. ovengensis			
An. somalicus			
An. aruni			
An. confusus			
An. funestus			
An. funestus-like			
An. longipalpis type C			
An. parensis			
An. vaneedeni			
An. brucei			
An. fuscivenosus			
An. rivulorum			
An. rivulorum-like			
An. leesoni			
An. moucheti			
An. bervoetsi			

**First Author**	**Coluzzi**	**Coluzzi**	**Collins**
**Year of publication**	**1968**	**1969**	**1987**
**Technique**	Cytogenetics: salivary glands	Cytogenetics	RFLP: DNA probe
**Species**			
An. amharicus			
An. arabiensis	1	1	1
An. bwambae			
An. coluzzii			
An. gambiae s.s.			
An. coluzzii/gambiae	1	1	1
An. melas		1	1
An. merus		1	
An. quadriannulatus	1	1	
An. nili			
An. carnevalei			
An. ovengensis			
An. somalicus			
An. aruni			
An. confusus			
An. funestus			
An. funestus-like			
An. longipalpis type C			
An. parensis			
An. vaneedeni			
An. brucei			
An. fuscivenosus			
An. rivulorum			
An. rivulorum-like			
An. leesoni			
An. moucheti			
An. bervoetsi			

**First Author**	**Paskewitz**	**Gentile**	**Van Rensburg**	**Townson**	**Charalambous**
**Year of publication**	**1990**	**2001**	**1996**	**1994**	**1999**
**Technique**	PCR: rDNA IGS	PCR: rDNA ITS	PCR: rDNA IGS	PCR: rDNA IGS	PCR: rDNA IGS
**Species**					
An. amharicus					
An. arabiensis	1		1		
An. bwambae				1	1
An. coluzzii		1			
An. gambiae s.s.		1			
An. coluzzii/gambiae	1		1		1
An. melas			1		
An. merus			1		
An. quadriannulatus			1		
An. nili					
An. carnevalei					
An. ovengensis					
An. somalicus					
An. aruni					
An. confusus					
An. funestus					
An. funestus-like					
An. longipalpis type C					
An. parensis					
An. vaneedeni					
An. brucei					
An. fuscivenosus					
An. rivulorum					
An. rivulorum-like					
An. leesoni					
An. moucheti					
An. bervoetsi					

**First Author**	**Miles**	**Gale**	**Gale**	**Coluzzi**
**Year of publication**	**1978**	**1987**	**1987**	**1978**
**Technique**	Electrophoresis: allozymes	DNA probes	DNA probes	Cytogenetics
**Species**				
An. amharicus				
An. arabiensis		1	1	1
An. bwambae	1			1
An. coluzzii				
An. gambiae s.s.				
An. coluzzii/gambiae		1	1	1
An. melas	1	1	1	1
An. merus	1	1	1	1
An. quadriannulatus	1		1	1
An. nili				
An. carnevalei				
An. ovengensis				
An. somalicus				
An. aruni				
An. confusus				
An. funestus				
An. funestus-like				
An. longipalpis type C				
An. parensis				
An. vaneedeni				
An. brucei				
An. fuscivenosus				
An. rivulorum				
An. rivulorum-like				
An. leesoni				
An. moucheti				
An. bervoetsi				

First Author	Carlson	Gatti	Frizzi
**Year of publication**	**1980**	**1977**	**1956**
**Technique**	Gas chromatography	Fluorescence banding	Cytogenetics: salivary glands
**Species**			
An. amharicus			
An. arabiensis	1	1	
An. bwambae			
An. coluzzii			
An. gambiae s.s.			
An. coluzzii/gambiae	1	1	1
An. melas			
An. merus			
An. quadriannulatus			
An. nili			
An. carnevalei			
An. ovengensis			
An. somalicus			
An. aruni			
An. confusus			
An. funestus			
An. funestus-like			
An. longipalpis type C			
An. parensis			
An. vaneedeni			
An. brucei			
An. fuscivenosus			
An. rivulorum			
An. rivulorum-like			
An. leesoni			
An. moucheti			
An. bervoetsi			

First Author	Fontenille	Paskewitz	Taylor	Wilkerson	Davidson
**Year of publication**	**1993**	**1993**	**1993**	**1993**	**1973**
**Technique**	PCR: rDNA IGS	PCR	RFLP & PCR	PCR-RAPD:	Cytogenetics
**Species**					
An. amharicus					
An. arabiensis	1	1	1	1	
An. bwambae					1
An. coluzzii					
An. gambiae s.s.					
An. coluzzii/gambiae	1		1	1	
An. melas					
An. merus		1	1		
An. quadriannulatus		1			
An. nili					
An. carnevalei					
An. ovengensis					
An. somalicus					
An. aruni					
An. confusus					
An. funestus					
An. funestus-like					
An. longipalpis type C					
An. parensis					
An. vaneedeni					
An. brucei					
An. fuscivenosus					
An. rivulorum					
An. rivulorum-like					
An. leesoni					
An. moucheti					
An. bervoetsi					

First Author	Coluzzi	Cornel	Hill	Collins
**Year of publication**	**1968**	**1997**	**1991**	**1988**
**Technique**	Cytogenetics: ovarian nurse cells	PCR: rDNA IGS	DNA probes	DNA probes
**Species**				
An. amharicus				
An. arabiensis	1	1	1	1
An. bwambae				
An. coluzzii				
An. gambiae s.s.				
An. coluzzii/gambiae	1	1	1	1
An. melas		1	1	1
An. merus		1	1	1
An. quadriannulatus		1		1
An. nili				
An. carnevalei				
An. ovengensis				
An. somalicus				
An. aruni				
An. confusus				
An. funestus				
An. funestus-like				
An. longipalpis type C				
An. parensis				
An. vaneedeni				
An. brucei				
An. fuscivenosus				
An. rivulorum				
An. rivulorum-like				
An. leesoni				
An. moucheti				
An. bervoetsi				

**First Author**	**Besansky**	**Hamilton**	**Kengne**	**Kengne**	
**Year of publication**	**2006**	**1983**	**2007**	**2003**	
**Technique**	PCR: rDNA IGS	CHC analysis	PCR: rDNA ITS1	PCR: rDNA ITS2	
**Species**					
An. amharicus					
An. arabiensis	1	1			
An. bwambae	1				
An. coluzzii					
An. gambiae s.s.					
An. coluzzii/gambiae	1	1			
An. melas	1	1			
An. merus	1				
An. quadriannulatus					
An. nili				1	
An. carnevalei				1	
An. ovengensis				1	
An. somalicus				1	
An. aruni					
An. confusus					
An. funestus					
An. funestus-like					
An. longipalpis type C					
An. parensis					
An. vaneedeni					
An. brucei					
An. fuscivenosus					
An. rivulorum					
An. rivulorum-like					
An. leesoni					
An. moucheti			1		
An. bervoetsi			1		

First Author	Koekemoer	Garros	Cohuet	Spillings
**Year of publication**	**2002**	**2004**	**2003**	**2009**
**Technique**	PCR: rDNA ITS2	PCR: rDNA ITS2	PCR: rDNA ITS2	PCR: rDNA ITS2
**Species**				
An. amharicus				
An. arabiensis				
An. bwambae				
An. coluzzii				
An. gambiae s.s.				
An. coluzzii/gambiae				
An. melas				
An. merus				
An. quadriannulatus				
An. nili				
An. carnevalei				
An. ovengensis				
An. somalicus				
An. aruni				
An. confusus				
An. funestus	1	1	1	1
An. funestus-like				1
An. longipalpis type C				
An. parensis	1	1	1	1
An. vaneedeni	1	1	1	1
An. brucei				
An. fuscivenosus				
An. rivulorum	1	1	1	1
An. rivulorum-like			1	
An. leesoni	1	1	1	1
An. moucheti				
An. bervoetsi				

**First Author**	**Garros**	**Green**
**Year of publication**	**2005**	**1979**
**Technique**	Sequencing: rDNA D3 & mtDNA COII	Cytogenetics: ovarian nurse cells
**Species**		
An. amharicus		
An. arabiensis		
An. bwambae		
An. coluzzii		
An. gambiae s.s.		
An. coluzzii/gambiae		
An. melas		
An. merus		
An. quadriannulatus		
An. nili		
An. carnevalei		
An. ovengensis		
An. somalicus		
An. aruni		
An. confusus		
An. funestus	1	1
An. funestus-like		
An. longipalpis type C		
An. parensis	1	1
An. vaneedeni	1	
An. brucei		
An. fuscivenosus		
An. rivulorum	1	
An. rivulorum-like	1	
An. leesoni	1	
An. moucheti		
An. bervoetsi		

**First Author**	**Choi**	**Garros**	
**Year of publication**	**2010**	**2004**	
**Technique**	PCR-RFLP: rDNA ITS2	PCR-RFLP: ITS2 & D3	
**Species**			
An. amharicus			
An. arabiensis			
An. bwambae			
An. coluzzii			
An. gambiae s.s.			
An. coluzzii/gambiae			
An. melas			
An. merus			
An. quadriannulatus			
An. nili			
An. carnevalei			
An. ovengensis			
An. somalicus			
An. aruni			
An. confusus			
An. funestus	1	1	
An. funestus-like	1		
An. longipalpis type C	1		
An. parensis		1	
An. vaneedeni	1	1	
An. brucei			
An. fuscivenosus			
An. rivulorum	1	1	
An. rivulorum-like			
An. leesoni	1	1	
An. moucheti			
An. bervoetsi			

**First Author**	**Green**	**Koekemoer**	**Koekemoer**
**Year of publication**	**1982**	**1998**	**1999**
**Technique**	Cytogenetics: salivary glands and nurse cells	PCR-RFLP: rDNA D3	PCR-SSCP
**Species**			
An. amharicus			
An. arabiensis			
An. bwambae			
An. coluzzii			
An. gambiae s.s.			
An. coluzzii/gambiae			
An. melas			
An. merus			
An. quadriannulatus			
An. nili			
An. carnevalei			
An. ovengensis			
An. somalicus			
An. aruni			
An. confusus	1		
An. funestus	1	1	1
An. funestus-like			
An. longipalpis type C			
An. parensis	1		
An. vaneedeni	1	1	
An. brucei			
An. fuscivenosus	1		
An. rivulorum	1		1
An. rivulorum-like			
An. leesoni	1		1
An. moucheti			
An. bervoetsi			

First Author	Hackett	Koekemoer	Chen
**Year of publication**	**2000**	**2009**	**2003**
**Technique**	PCR: rDNA ITS2	PCR: rDNA ITS2	Sequencing: rDNA D3
**Species**			
An. amharicus			
An. arabiensis			
An. bwambae			
An. coluzzii			
An. gambiae s.s.			
An. coluzzii/gambiae			
An. melas			
An. merus			
An. quadriannulatus			
An. nili			
An. carnevalei			
An. ovengensis			
An. somalicus			
An. aruni			
An. confusus			
An. funestus	1	1	1
An. funestus-like			
An. longipalpis type C		1	
An. parensis		1	
An. vaneedeni		1	
An. brucei			
An. fuscivenosus			
An. rivulorum	1	1	
An. rivulorum-like			
An. leesoni		1	1
An. moucheti			
An. bervoetsi			

First Author	Garros	Vezenegho	
Year of publication	2005	2009	
Technique	Sequencing: rDNA ITS2, D3 & mtDNA COI	PCR-TaqMan	
Species			
An. amharicus			
An. arabiensis			
An. bwambae			
An. coluzzii			
An. gambiae s.s.			
An. coluzzii/gambiae			
An. melas			
An. merus			
An. quadriannulatus			
An. nili			
An. carnevalei			
An. ovengensis			
An. somalicus			
An. aruni			
An. confusus			
An. funestus	1	1	
An. funestus-like			
An. longipalpis type C			
An. parensis	1	1	
An. vaneedeni	1	1	
An. brucei			
An. fuscivenosus			
An. rivulorum	1	1	
An. rivulorum-like			
An. leesoni	1	1	
An. moucheti			
An. bervoetsi			

First Author	Weeto	Singh
**Year of publication**	**2004**	**2010**
**Technique**	PCR-SSCP & PCR: rDNA ITS2	Sequencing: rDNA ITS & D3
**Species**		
An. amharicus		
An. arabiensis		
An. bwambae		
An. coluzzii		
An. gambiae s.s.		
An. coluzzii/gambiae		
An. melas		
An. merus		
An. quadriannulatus		
An. nili		
An. carnevalei		
An. ovengensis		
An. somalicus		
An. aruni		
An. confusus		
An. funestus	1	
An. funestus-like		
An. longipalpis type C		
An. parensis	1	
An. vaneedeni	1	
An. brucei		
An. fuscivenosus		
An. rivulorum	1	
An. rivulorum-like		
An. leesoni	1	
An. moucheti		
An. bervoetsi		

**First Author**	**Koekemoer**
**Year of publication**	**2002**
**Technique**	PCR-SSCP & PCR: rDNA ITS2
**Species**	
An. amharicus	
An. arabiensis	
An. bwambae	
An. coluzzii	
An. gambiae s.s.	
An. coluzzii/gambiae	
An. melas	
An. merus	
An. quadriannulatus	
An. nili	
An. carnevalei	
An. ovengensis	
An. somalicus	
An. aruni	
An. confusus	
An. funestus	1
An. funestus-like	
An. longipalpis type C	
An. parensis	1
An. vaneedeni	1
An. brucei	
An. fuscivenosus	
An. rivulorum	1
An. rivulorum-like	
An. leesoni	1
An. moucheti	
An. bervoetsi	
